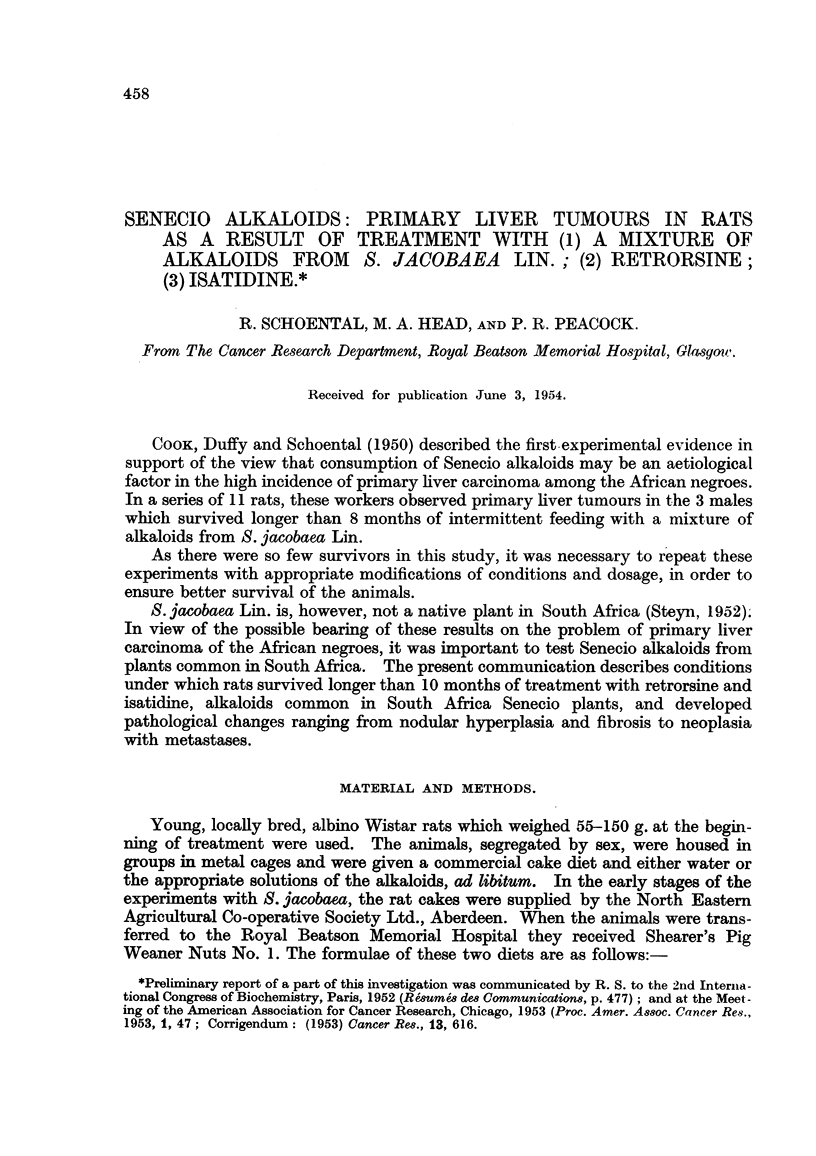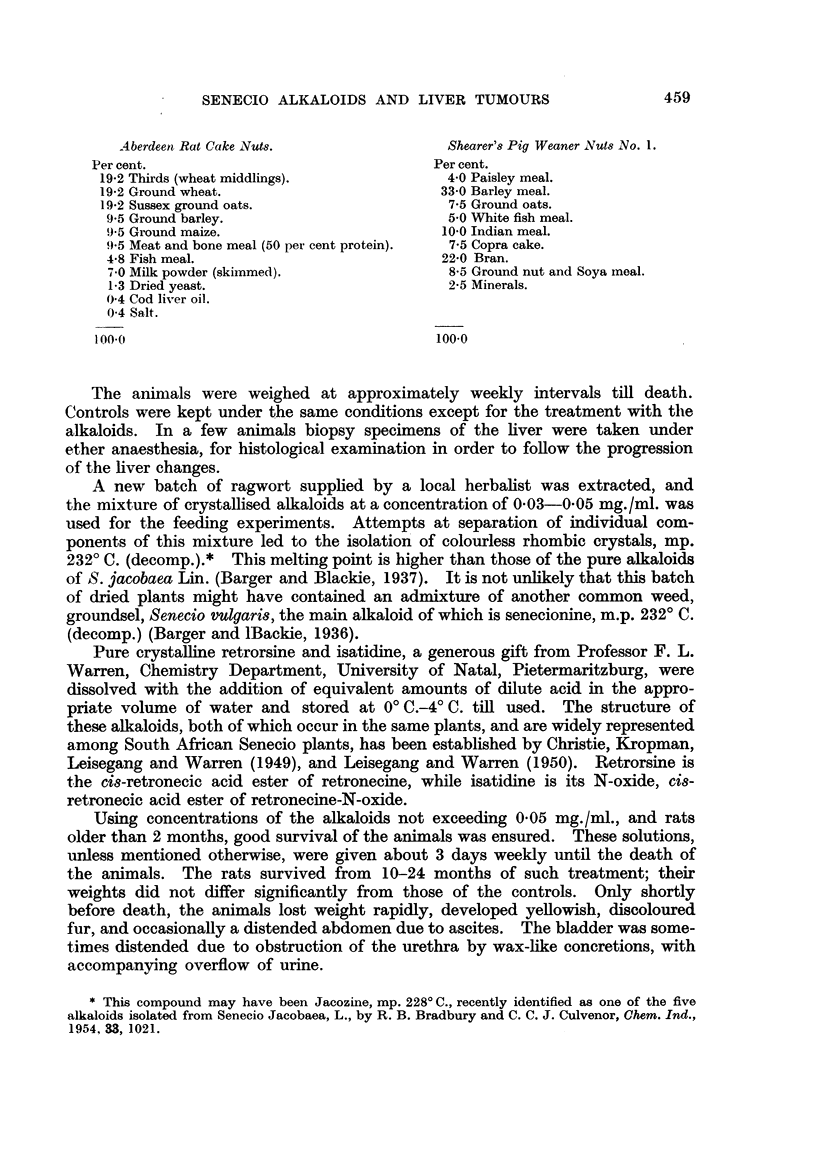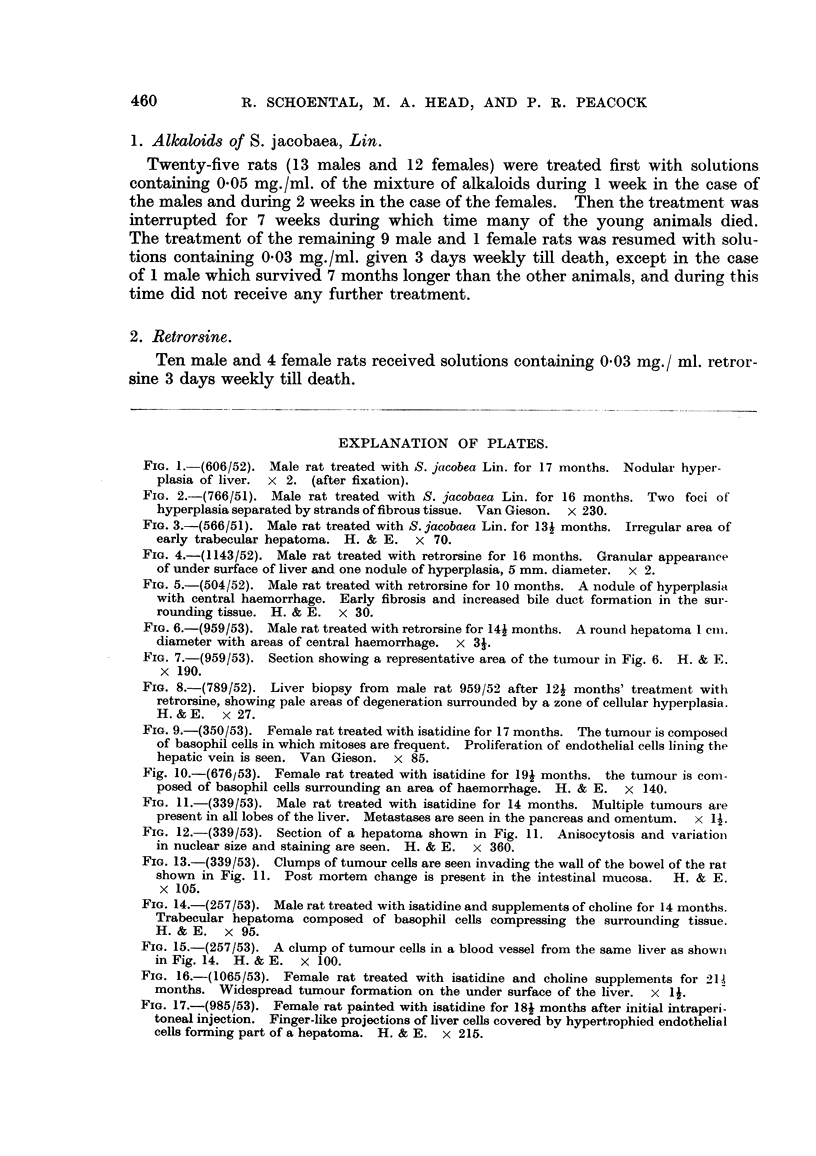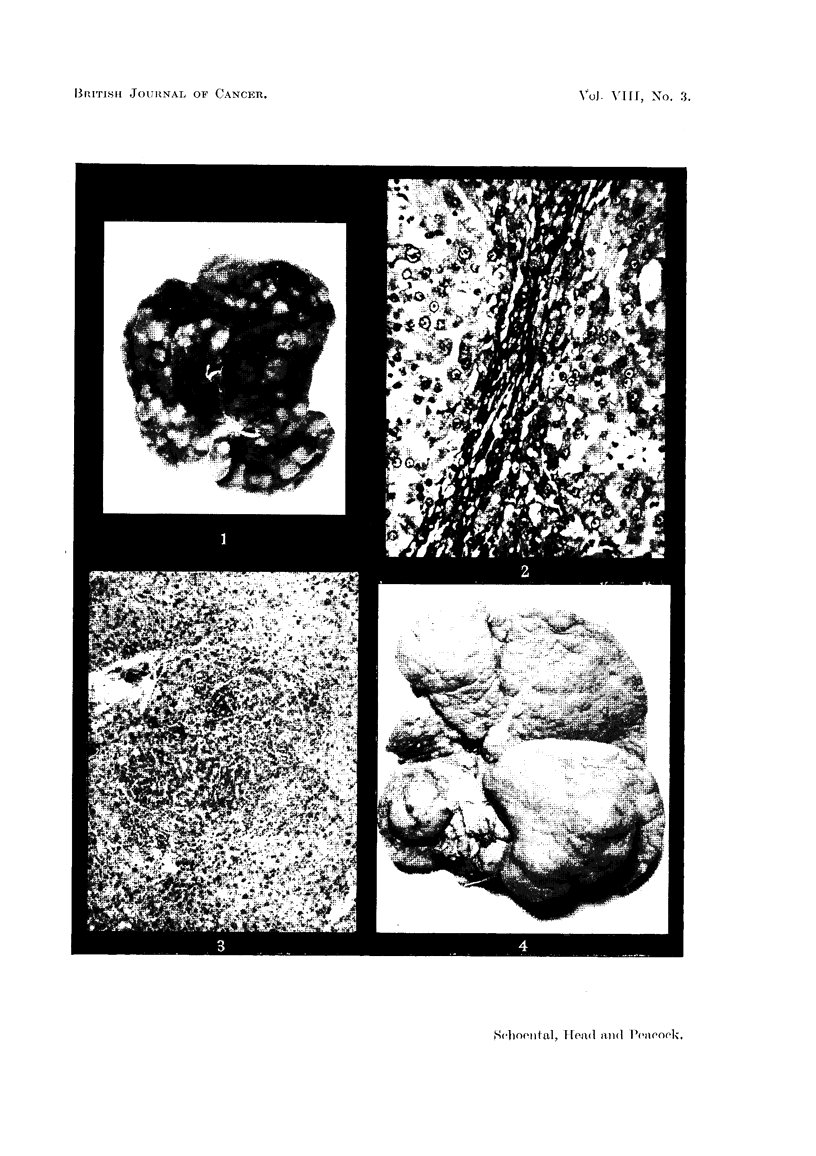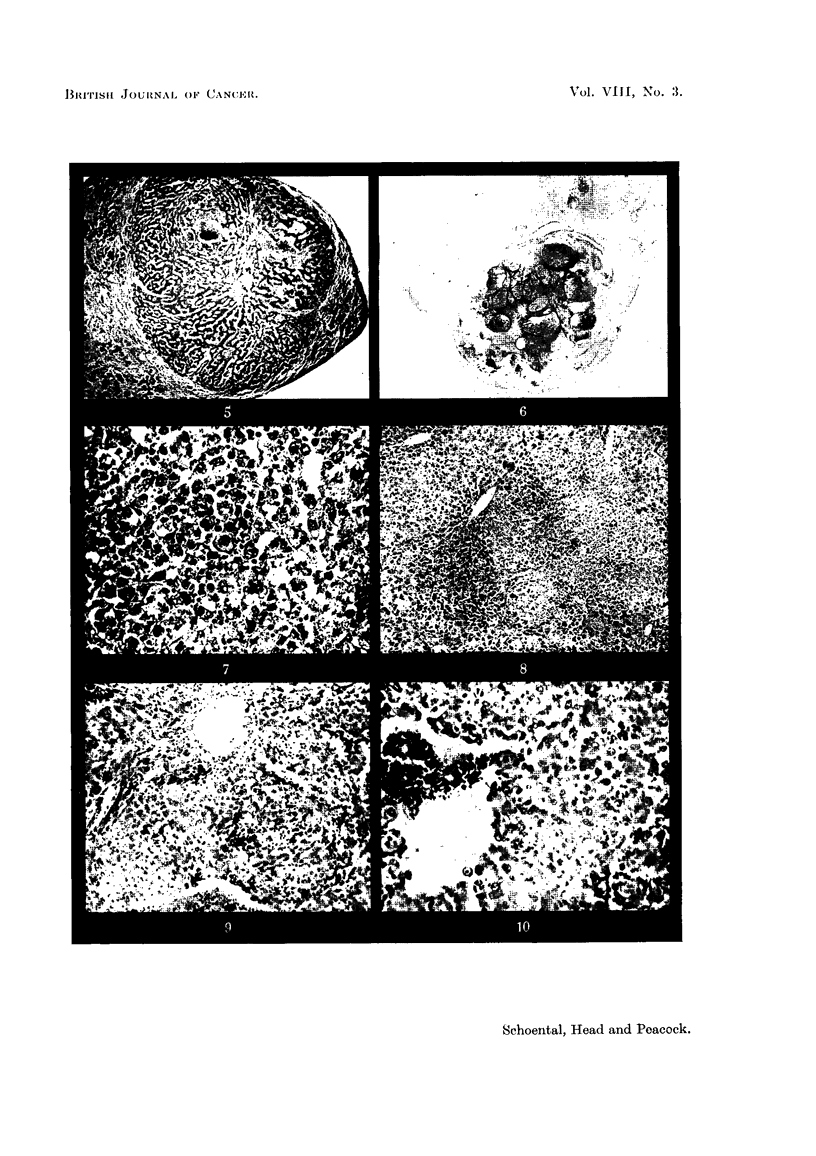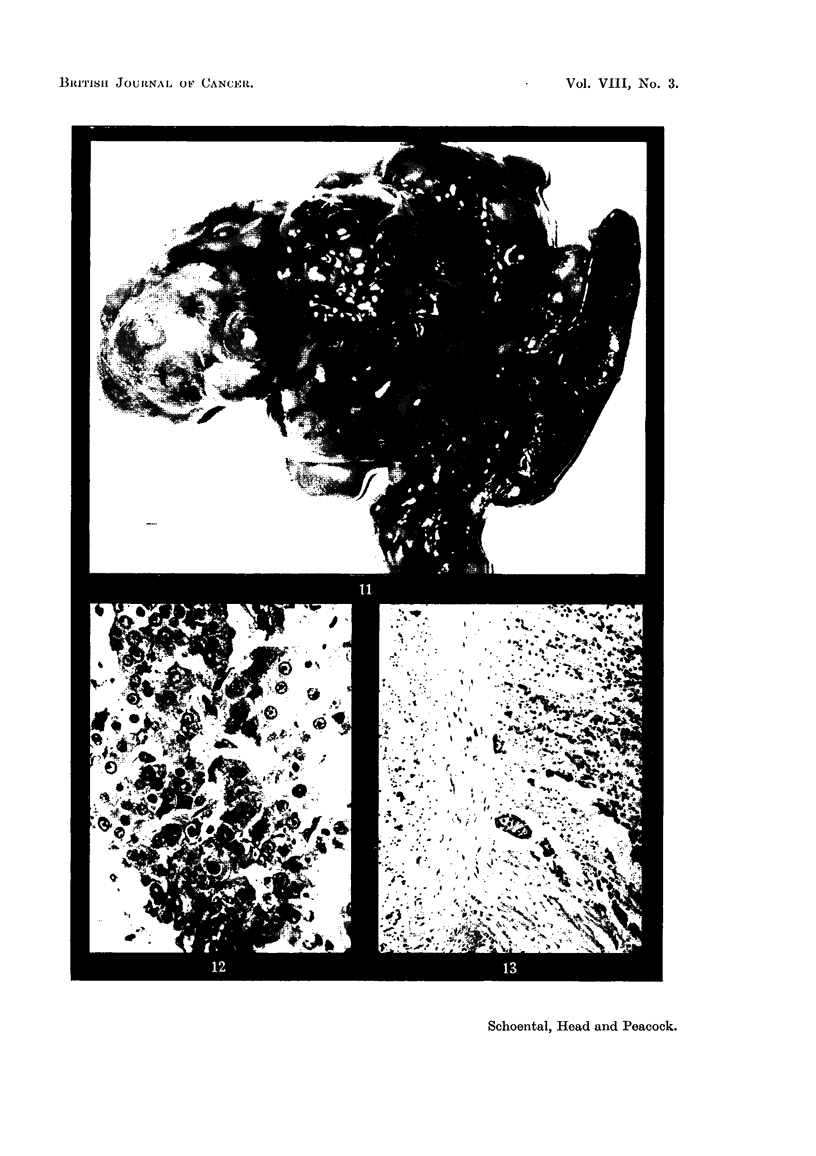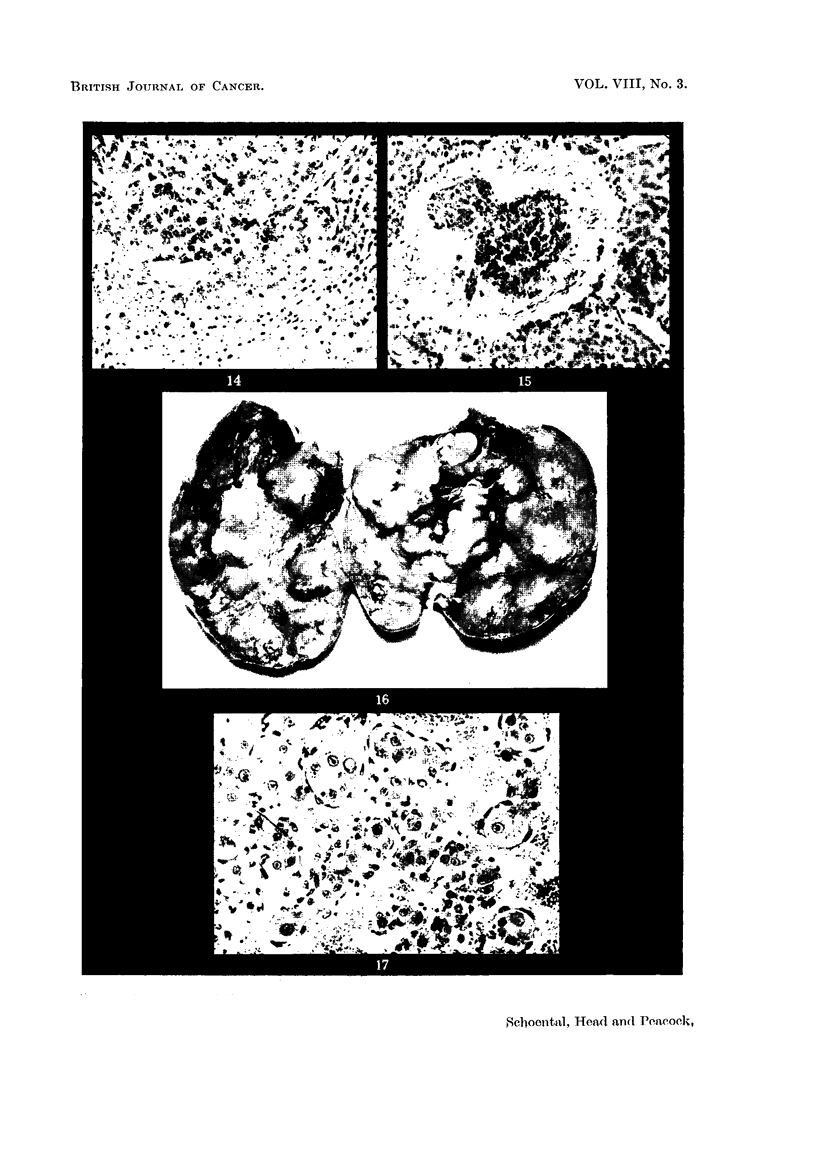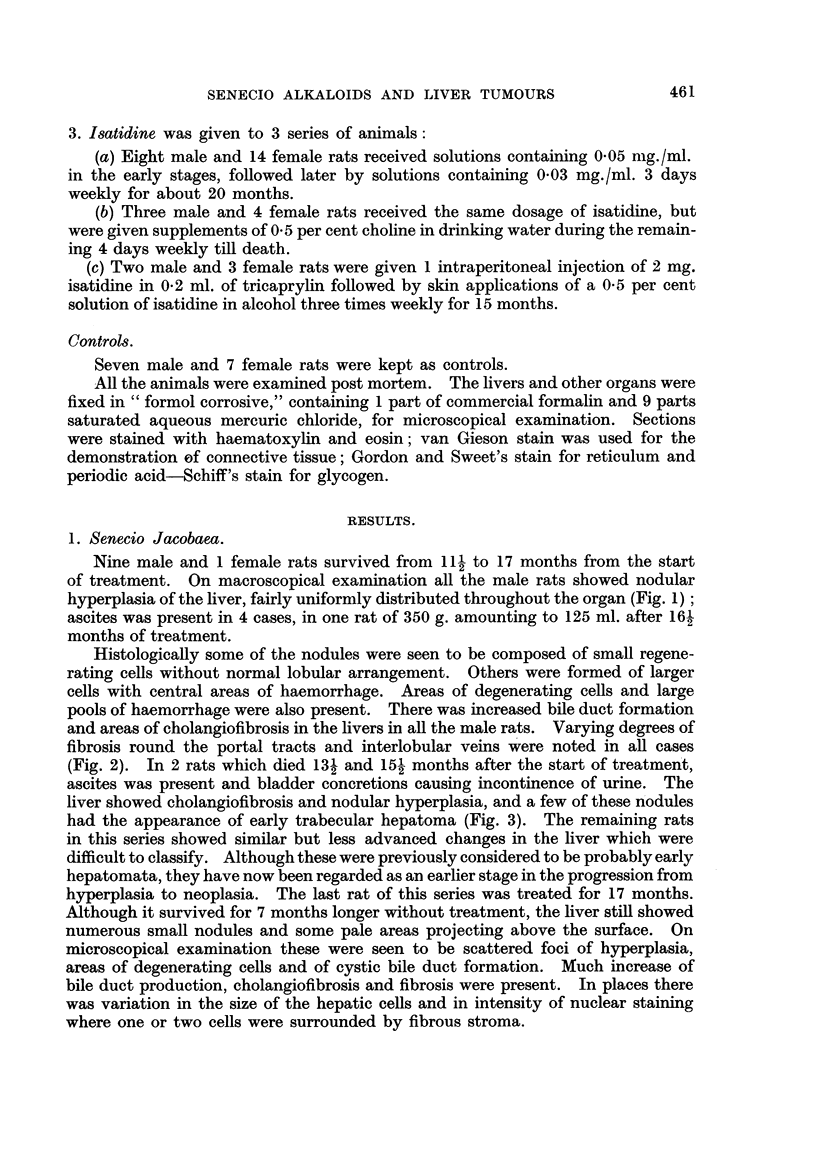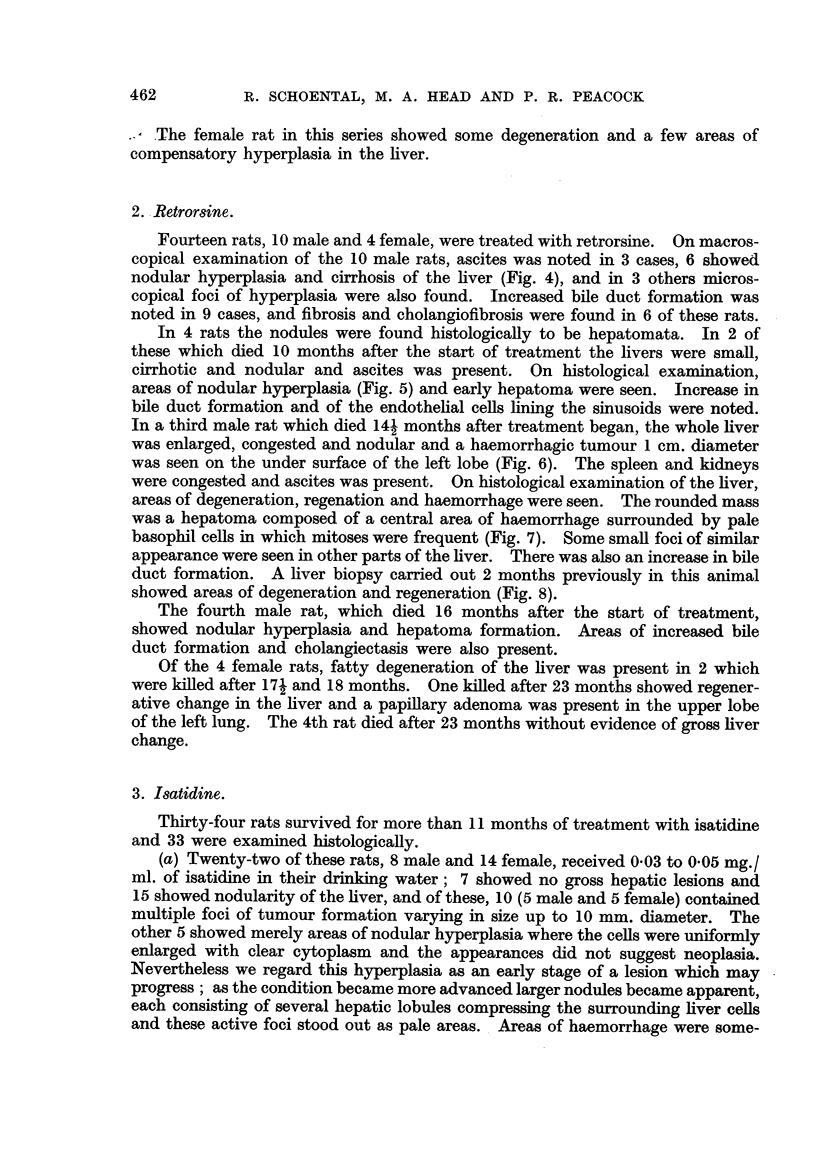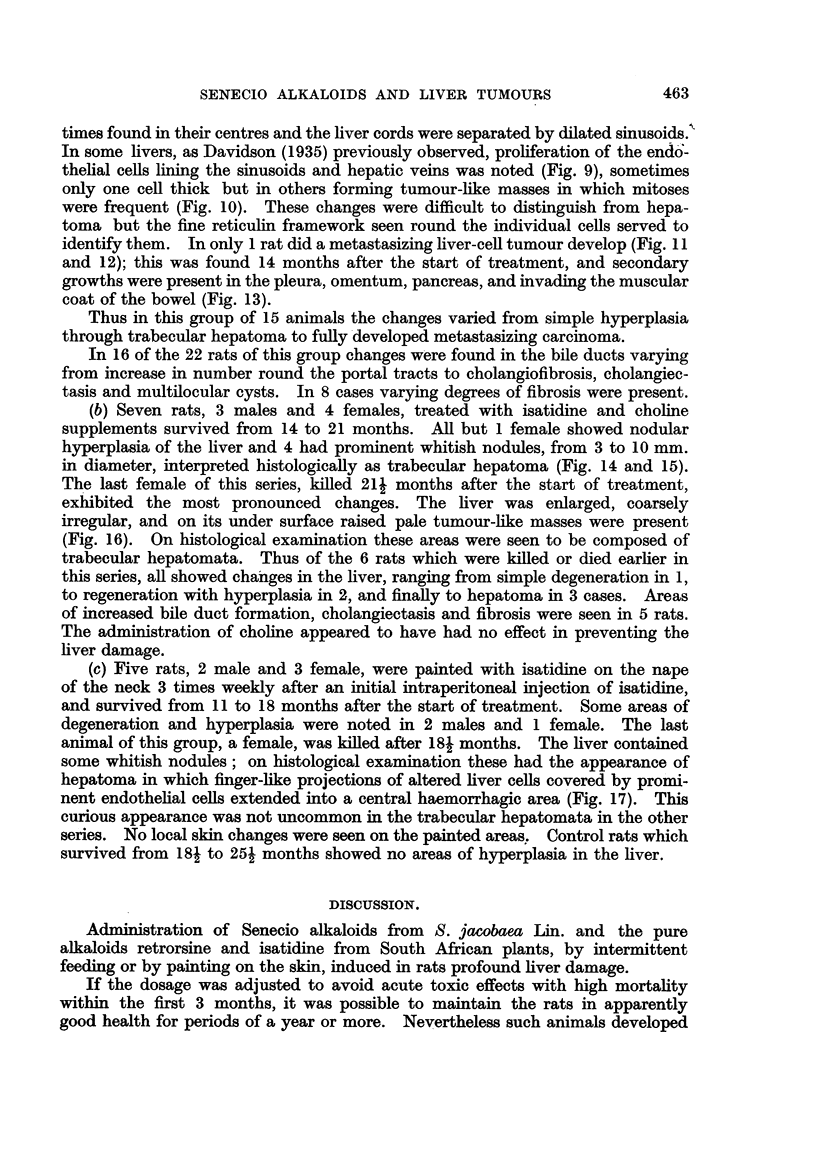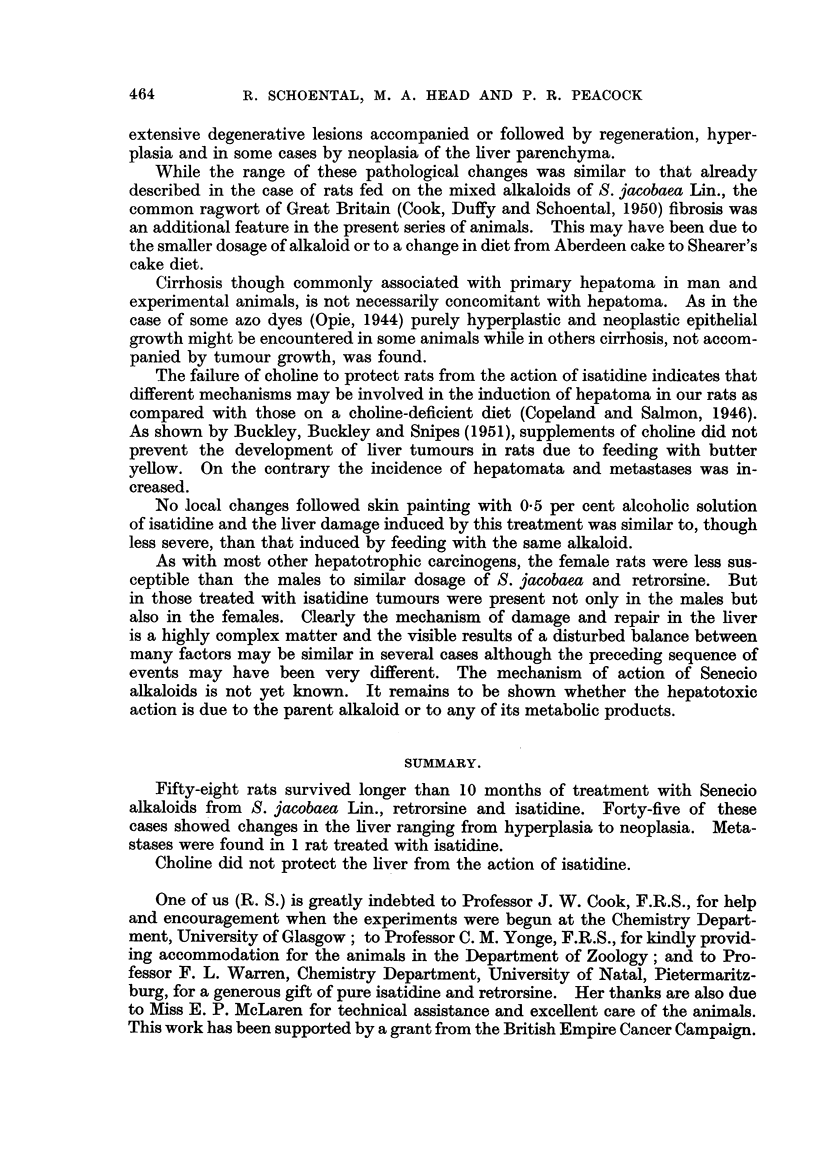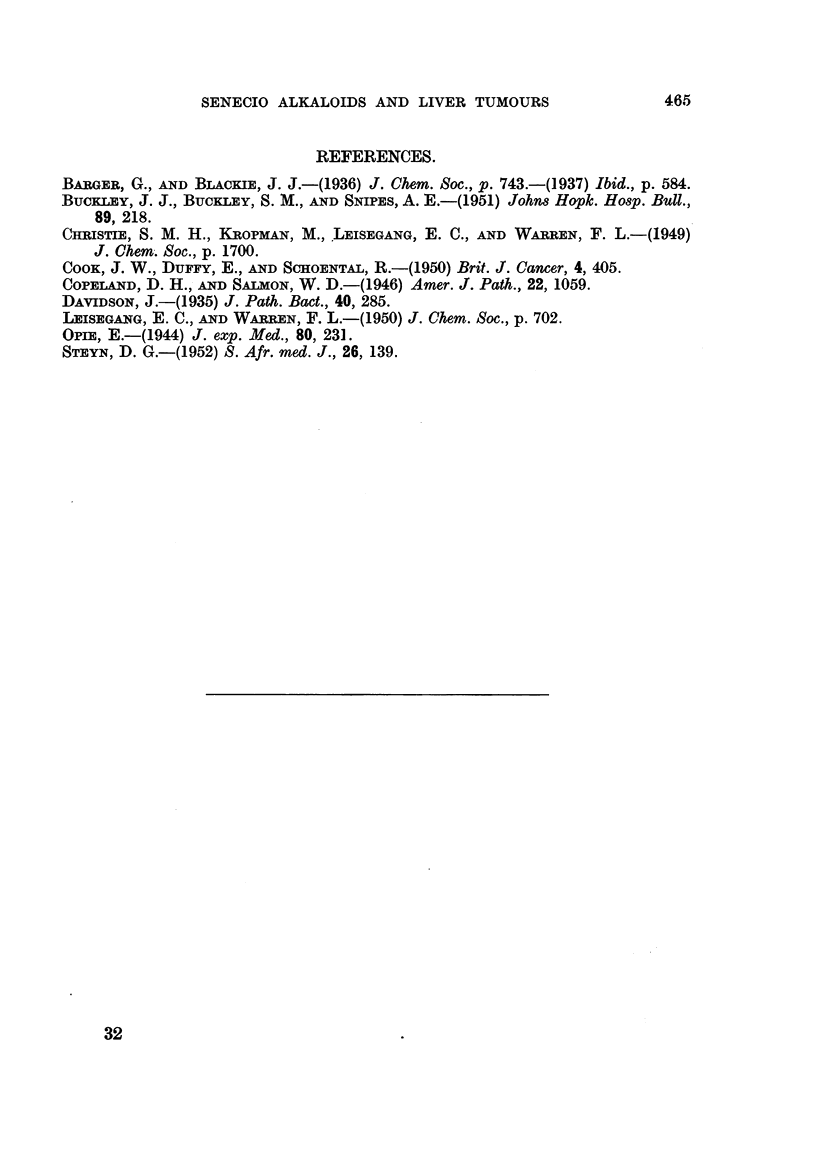# Senecio Alkaloids: Primary Liver Tumours in Rats as a Result of Treatment with (1) A Mixture of Alkaloids from S. Jacobaea Lin.; (2) Retrorsine; (3) Isatidine*

**DOI:** 10.1038/bjc.1954.49

**Published:** 1954-09

**Authors:** R. Schoental, M. A. Head, P. R. Peacock

## Abstract

**Images:**


### SENECIO ALKALOIDS: PRIMARY LIVER TUMOURS IN RATS AS A RESULT OF TREATMENT WITH (1) A MIXTURE OF ALKALOIDS FROM *S. JACOBAEA* LIN.; (2) RETRORSINE; (3) ISATIDINE.*

R. SCHOENTAL, M. A. HEAD, AND P. R. PEACOCK.

*From The Cancer Research Department, Royal Beatson Memorial Hospital, Glasgow.*

Received for publication June 3, 1954.

COOK, Duffy and Schoental (1950) described the first experimental evidence in support of the view that consumption of Senecio alkaloids may be an aetiological factor in the high incidence of primary liver carcinoma among the African negroes. In a series of 11 rats, these workers observed primary liver tumours in the 3 males which survived longer than 8 months of intermittent feeding with a mixture of alkaloids from *S. jacobaea* Lin.

As there were so few survivors in this study, it was necessary to repeat these experiments with appropriate modifications of conditions and dosage, in order to ensure better survival of the animals.

*S. jacobaea* Lin. is, however, not a native plant in South Africa (Steyn, 1952). In view of the possible bearing of these results on the problem of primary liver carcinoma of the African negroes, it was important to test Senecio alkaloids from plants common in South Africa. The present communication describes conditions under which rats survived longer than 10 months of treatment with retrorsine and isatidine, alkaloids common in South Africa Senecio plants, and developed pathological changes ranging from nodular hyperplasia and fibrosis to neoplasia with metastases.

### MATERIAL AND METHODS.

Young, locally bred, albino Wistar rats which weighed 55–150 g. at the beginning of treatment were used. The animals, segregated by sex, were housed in groups in metal cages and were given a commercial cake diet and either water or the appropriate solutions of the alkaloids, *ad libitum.* In the early stages of the experiments with *S. jacobaea*, the rat cakes were supplied by the North Eastern Agricultural Co-operative Society Ltd., Aberdeen. When the animals were transferred to the Royal Beatson Memorial Hospital they received Shearer's Pig Weaner Nuts No. 1. The formulae of these two diets are as follows:—

*Preliminary report of a part of this investigation was communicated by R. S. to the 2nd International Congress of Biochemistry, Paris, 1952 (*Résumés des Communications*, p. 477); and at the Meeting of the American Association for Cancer Research, Chicago, 1953 (*Proc. Amer. Assoc. Cancer Res.*, 1953, **1**, 47; Corrigendum: (1953) *Cancer Res.*, **13**, 616.

*Aberdeen Rat Cake Nuts.*

| Per cent. | |
|---|---|
| 19·2 | Thirds (wheat middlings). |
| 19·2 | Ground wheat. |
| 19·2 | Sussex ground oats. |
| 9·5 | Ground barley. |
| 9·5 | Ground maize. |
| 9·5 | Meat and bone meal (50 per cent protein). |
| 4·8 | Fish meal. |
| 7·0 | Milk powder (skimmed). |
| 1·3 | Dried yeast. |
| 0·4 | Cod liver oil. |
| 0·4 | Salt. |
| 100·0 | |

*Shearer's Pig Weaner Nuts No.* 1.

| Per cent. | |
|---|---|
| 4·0 | Paisley meal. |
| 33·0 | Barley meal. |
| 7·5 | Ground oats. |
| 5·0 | White fish meal. |
| 10·0 | Indian meal. |
| 7·5 | Copra cake. |
| 22·0 | Bran. |
| 8·5 | Ground nut and Soya meal. |
| 2·5 | Minerals. |
| 100·0 | |

The animals were weighed at approximately weekly intervals till death. Controls were kept under the same conditions except for the treatment with the alkaloids. In a few animals biopsy specimens of the liver were taken under ether anaesthesia, for histological examination in order to follow the progression of the liver changes.

A new batch of ragwort supplied by a local herbalist was extracted, and the mixture of crystallised alkaloids at a concentration of 0·03—0·05 mg./ml. was used for the feeding experiments. Attempts at separation of individual components of this mixture led to the isolation of colourless rhombic crystals, mp. 232° C. (decomp.).* This melting point is higher than those of the pure alkaloids of *S. jacobaea* Lin. (Barger and Blackie, 1937). It is not unlikely that this batch of dried plants might have contained an admixture of another common weed, groundsel, *Senecio vulgaris*, the main alkaloid of which is senecionine, m.p. 232° C. (decomp.) (Barger and lBackie, 1936).

Pure crystalline retrorsine and isatidine, a generous gift from Professor F. L. Warren, Chemistry Department, University of Natal, Pietermaritzburg, were dissolved with the addition of equivalent amounts of dilute acid in the appropriate volume of water and stored at 0° C.–4° C. till used. The structure of these alkaloids, both of which occur in the same plants, and are widely represented among South African Senecio plants, has been established by Christie, Kropman, Leisegang and Warren (1949), and Leisegang and Warren (1950). Retrorsine is the *cis*-retronecic acid ester of retronecine, while isatidine is its N-oxide, *cis*-retronecic acid ester of retronecine-N-oxide.

Using concentrations of the alkaloids not exceeding 0·05 mg./ml., and rats older than 2 months, good survival of the animals was ensured. These solutions, unless mentioned otherwise, were given about 3 days weekly until the death of the animals. The rats survived from 10–24 months of such treatment; their weights did not differ significantly from those of the controls. Only shortly before death, the animals lost weight rapidly, developed yellowish, discoloured fur, and occasionally a distended abdomen due to ascites. The bladder was sometimes distended due to obstruction of the urethra by wax-like concretions, with accompanying overflow of urine.

* This compound may have been Jacozine, mp. 228° C., recently identified as one of the five alkaloids isolated from Senecio Jacobaea, L., by R. B. Bradbury and C. C. J. Culvenor, *Chem. Ind.*, 1954, **33**, 1021.

1. *Alkaloids of* S. jacobaea, *Lin.*

Twenty-five rats (13 males and 12 females) were treated first with solutions containing 0·05 mg./ml. of the mixture of alkaloids during 1 week in the case of the males and during 2 weeks in the case of the females. Then the treatment was interrupted for 7 weeks during which time many of the young animals died. The treatment of the remaining 9 male and 1 female rats was resumed with solutions containing 0·03 mg./ml. given 3 days weekly till death, except in the case of 1 male which survived 7 months longer than the other animals, and during this time did not receive any further treatment.

2. *Retrorsine.*

Ten male and 4 female rats received solutions containing 0·03 mg./ ml. retrorsine 3 days weekly till death.

---

EXPLANATION OF PLATES.

FIG. 1.—(606/52). Male rat treated with *S. jacobea* Lin. for 17 months. Nodular hyperplasia of liver. × 2. (after fixation).

FIG. 2.—(766/51). Male rat treated with *S. jacobaea* Lin. for 16 months. Two foci of hyperplasia separated by strands of fibrous tissue. Van Gieson. × 230.

FIG. 3.—(566/51). Male rat treated with *S. jacobaea* Lin. for 13½ months. Irregular area of early trabecular hepatoma. H. & E. × 70.

FIG. 4.—(1143/52). Male rat treated with retrorsine for 16 months. Granular appearance of under surface of liver and one nodule of hyperplasia, 5 mm. diameter. × 2.

FIG. 5.—(504/52). Male rat treated with retrorsine for 10 months. A nodule of hyperplasia with central haemorrhage. Early fibrosis and increased bile duct formation in the surrounding tissue. H. & E. × 30.

FIG. 6.—(959/53). Male rat treated with retrorsine for 14½ months. A round hepatoma 1 cm. diameter with areas of central haemorrhage. × 3½.

FIG. 7.—(959/53). Section showing a representative area of the tumour in Fig. 6. H. & E. × 190.

FIG. 8.—(789/52). Liver biopsy from male rat 959/52 after 12½ months' treatment with retrorsine, showing pale areas of degeneration surrounded by a zone of cellular hyperplasia. H. & E. × 27.

FIG. 9.—(350/53). Female rat treated with isatidine for 17 months. The tumour is composed of basophil cells in which mitoses are frequent. Proliferation of endothelial cells lining the hepatic vein is seen. Van Gieson. × 85.

Fig. 10.—(676/53). Female rat treated with isatidine for 19½ months. the tumour is composed of basophil cells surrounding an area of haemorrhage. H. & E. × 140.

FIG. 11.—(339/53). Male rat treated with isatidine for 14 months. Multiple tumours are present in all lobes of the liver. Metastases are seen in the pancreas and omentum. × 1½.

FIG. 12.—(339/53). Section of a hepatoma shown in Fig. 11. Anisocytosis and variation in nuclear size and staining are seen. H. & E. × 360.

FIG. 13.—(339/53). Clumps of tumour cells are seen invading the wall of the bowel of the rat shown in Fig. 11. Post mortem change is present in the intestinal mucosa. H. & E. × 105.

FIG. 14.—(257/53). Male rat treated with isatidine and supplements of choline for 14 months. Trabecular hepatoma composed of basophil cells compressing the surrounding tissue. H. & E. × 95.

FIG. 15.—(257/53). A clump of tumour cells in a blood vessel from the same liver as shown in Fig. 14. H. & E. × 100.

FIG. 16.—(1065/53). Female rat treated with isatidine and choline supplements for 21½ months. Widespread tumour formation on the under surface of the liver. × 1½.

FIG. 17.—(985/53). Female rat painted with isatidine for 18½ months after initial intraperitoneal injection. Finger-like projections of liver cells covered by hypertrophied endothelial cells forming part of a hepatoma. H. & E. × 215.

1

2

3

4

Schoental, Head and Peacock.

5 6 7 8 9 10

Schoental, Head and Peacock.

11

12

13

Schoental, Head and Peacock.

14

15

16

17

3. *Isatidine* was given to 3 series of animals:

(*a*) Eight male and 14 female rats received solutions containing 0·05 mg./ml. in the early stages, followed later by solutions containing 0·03 mg./ml. 3 days weekly for about 20 months.

(*b*) Three male and 4 female rats received the same dosage of isatidine, but were given supplements of 0·5 per cent choline in drinking water during the remaining 4 days weekly till death.

(*c*) Two male and 3 female rats were given 1 intraperitoneal injection of 2 mg. isatidine in 0·2 ml. of tricaprylin followed by skin applications of a 0·5 per cent solution of isatidine in alcohol three times weekly for 15 months.

*Controls.*

Seven male and 7 female rats were kept as controls.

All the animals were examined post mortem. The livers and other organs were fixed in " formol corrosive," containing 1 part of commercial formalin and 9 parts saturated aqueous mercuric chloride, for microscopical examination. Sections were stained with haematoxylin and eosin; van Gieson stain was used for the demonstration of connective tissue; Gordon and Sweet's stain for reticulum and periodic acid—Schiff's stain for glycogen.

### RESULTS.

1. *Senecio Jacobaea.*

Nine male and 1 female rats survived from 11½ to 17 months from the start of treatment. On macroscopical examination all the male rats showed nodular hyperplasia of the liver, fairly uniformly distributed throughout the organ (Fig. 1); ascites was present in 4 cases, in one rat of 350 g. amounting to 125 ml. after 16½ months of treatment.

Histologically some of the nodules were seen to be composed of small regenerating cells without normal lobular arrangement. Others were formed of larger cells with central areas of haemorrhage. Areas of degenerating cells and large pools of haemorrhage were also present. There was increased bile duct formation and areas of cholangiofibrosis in the livers in all the male rats. Varying degrees of fibrosis round the portal tracts and interlobular veins were noted in all cases (Fig. 2). In 2 rats which died 13½ and 15½ months after the start of treatment, ascites was present and bladder concretions causing incontinence of urine. The liver showed cholangiofibrosis and nodular hyperplasia, and a few of these nodules had the appearance of early trabecular hepatoma (Fig. 3). The remaining rats in this series showed similar but less advanced changes in the liver which were difficult to classify. Although these were previously considered to be probably early hepatomata, they have now been regarded as an earlier stage in the progression from hyperplasia to neoplasia. The last rat of this series was treated for 17 months. Although it survived for 7 months longer without treatment, the liver still showed numerous small nodules and some pale areas projecting above the surface. On microscopical examination these were seen to be scattered foci of hyperplasia, areas of degenerating cells and of cystic bile duct formation. Much increase of bile duct production, cholangiofibrosis and fibrosis were present. In places there was variation in the size of the hepatic cells and in intensity of nuclear staining where one or two cells were surrounded by fibrous stroma.

The female rat in this series showed some degeneration and a few areas of compensatory hyperplasia in the liver.

2. *Retrorsine.*

Fourteen rats, 10 male and 4 female, were treated with retrorsine. On macroscopical examination of the 10 male rats, ascites was noted in 3 cases, 6 showed nodular hyperplasia and cirrhosis of the liver (Fig. 4), and in 3 others microscopical foci of hyperplasia were also found. Increased bile duct formation was noted in 9 cases, and fibrosis and cholangiofibrosis were found in 6 of these rats.

In 4 rats the nodules were found histologically to be hepatomata. In 2 of these which died 10 months after the start of treatment the livers were small, cirrhotic and nodular and ascites was present. On histological examination, areas of nodular hyperplasia (Fig. 5) and early hepatoma were seen. Increase in bile duct formation and of the endothelial cells lining the sinusoids were noted. In a third male rat which died 14½ months after treatment began, the whole liver was enlarged, congested and nodular and a haemorrhagic tumour 1 cm. diameter was seen on the under surface of the left lobe (Fig. 6). The spleen and kidneys were congested and ascites was present. On histological examination of the liver, areas of degeneration, regenation and haemorrhage were seen. The rounded mass was a hepatoma composed of a central area of haemorrhage surrounded by pale basophil cells in which mitoses were frequent (Fig. 7). Some small foci of similar appearance were seen in other parts of the liver. There was also an increase in bile duct formation. A liver biopsy carried out 2 months previously in this animal showed areas of degeneration and regeneration (Fig. 8).

The fourth male rat, which died 16 months after the start of treatment, showed nodular hyperplasia and hepatoma formation. Areas of increased bile duct formation and cholangiectasis were also present.

Of the 4 female rats, fatty degeneration of the liver was present in 2 which were killed after 17½ and 18 months. One killed after 23 months showed regenerative change in the liver and a papillary adenoma was present in the upper lobe of the left lung. The 4th rat died after 23 months without evidence of gross liver change.

3. *Isatidine.*

Thirty-four rats survived for more than 11 months of treatment with isatidine and 33 were examined histologically.

(*a*) Twenty-two of these rats, 8 male and 14 female, received 0·03 to 0·05 mg./ml. of isatidine in their drinking water ; 7 showed no gross hepatic lesions and 15 showed nodularity of the liver, and of these, 10 (5 male and 5 female) contained multiple foci of tumour formation varying in size up to 10 mm. diameter. The other 5 showed merely areas of nodular hyperplasia where the cells were uniformly enlarged with clear cytoplasm and the appearances did not suggest neoplasia. Nevertheless we regard this hyperplasia as an early stage of a lesion which may progress ; as the condition became more advanced larger nodules became apparent, each consisting of several hepatic lobules compressing the surrounding liver cells and these active foci stood out as pale areas. Areas of haemorrhage were sometimes found in their centres and the liver cords were separated by dilated sinusoids. In some livers, as Davidson (1935) previously observed, proliferation of the endothelial cells lining the sinusoids and hepatic veins was noted (Fig. 9), sometimes only one cell thick but in others forming tumour-like masses in which mitoses were frequent (Fig. 10). These changes were difficult to distinguish from hepatoma but the fine reticulin framework seen round the individual cells served to identify them. In only 1 rat did a metastasizing liver-cell tumour develop (Fig. 11 and 12); this was found 14 months after the start of treatment, and secondary growths were present in the pleura, omentum, pancreas, and invading the muscular coat of the bowel (Fig. 13).

Thus in this group of 15 animals the changes varied from simple hyperplasia through trabecular hepatoma to fully developed metastasizing carcinoma.

In 16 of the 22 rats of this group changes were found in the bile ducts varying from increase in number round the portal tracts to cholangiofibrosis, cholangiectasis and multilocular cysts. In 8 cases varying degrees of fibrosis were present.

(*b*) Seven rats, 3 males and 4 females, treated with isatidine and choline supplements survived from 14 to 21 months. All but 1 female showed nodular hyperplasia of the liver and 4 had prominent whitish nodules, from 3 to 10 mm. in diameter, interpreted histologically as trabecular hepatoma (Fig. 14 and 15). The last female of this series, killed 21½ months after the start of treatment, exhibited the most pronounced changes. The liver was enlarged, coarsely irregular, and on its under surface raised pale tumour-like masses were present (Fig. 16). On histological examination these areas were seen to be composed of trabecular hepatomata. Thus of the 6 rats which were killed or died earlier in this series, all showed changes in the liver, ranging from simple degeneration in 1, to regeneration with hyperplasia in 2, and finally to hepatoma in 3 cases. Areas of increased bile duct formation, cholangiectasis and fibrosis were seen in 5 rats. The administration of choline appeared to have had no effect in preventing the liver damage.

(*c*) Five rats, 2 male and 3 female, were painted with isatidine on the nape of the neck 3 times weekly after an initial intraperitoneal injection of isatidine, and survived from 11 to 18 months after the start of treatment. Some areas of degeneration and hyperplasia were noted in 2 males and 1 female. The last animal of this group, a female, was killed after 18½ months. The liver contained some whitish nodules ; on histological examination these had the appearance of hepatoma in which finger-like projections of altered liver cells covered by prominent endothelial cells extended into a central haemorrhagic area (Fig. 17). This curious appearance was not uncommon in the trabecular hepatomata in the other series. No local skin changes were seen on the painted areas. Control rats which survived from 18½ to 25½ months showed no areas of hyperplasia in the liver.

### DISCUSSION.

Administration of Senecio alkaloids from *S. jacobaea* Lin. and the pure alkaloids retrorsine and isatidine from South African plants, by intermittent feeding or by painting on the skin, induced in rats profound liver damage.

If the dosage was adjusted to avoid acute toxic effects with high mortality within the first 3 months, it was possible to maintain the rats in apparently good health for periods of a year or more. Nevertheless such animals developed extensive degenerative lesions accompanied or followed by regeneration, hyperplasia and in some cases by neoplasia of the liver parenchyma.

While the range of these pathological changes was similar to that already described in the case of rats fed on the mixed alkaloids of *S. jacobaea* Lin., the common ragwort of Great Britain (Cook, Duffy and Schoental, 1950) fibrosis was an additional feature in the present series of animals. This may have been due to the smaller dosage of alkaloid or to a change in diet from Aberdeen cake to Shearer's cake diet.

Cirrhosis though commonly associated with primary hepatoma in man and experimental animals, is not necessarily concomitant with hepatoma. As in the case of some azo dyes (Opie, 1944) purely hyperplastic and neoplastic epithelial growth might be encountered in some animals while in others cirrhosis, not accompanied by tumour growth, was found.

The failure of choline to protect rats from the action of isatidine indicates that different mechanisms may be involved in the induction of hepatoma in our rats as compared with those on a choline-deficient diet (Copeland and Salmon, 1946). As shown by Buckley, Buckley and Snipes (1951), supplements of choline did not prevent the development of liver tumours in rats due to feeding with butter yellow. On the contrary the incidence of hepatomata and metastases was increased.

No local changes followed skin painting with 0·5 per cent alcoholic solution of isatidine and the liver damage induced by this treatment was similar to, though less severe, than that induced by feeding with the same alkaloid.

As with most other hepatotrophic carcinogens, the female rats were less susceptible than the males to similar dosage of *S. jacobaea* and retrorsine. But in those treated with isatidine tumours were present not only in the males but also in the females. Clearly the mechanism of damage and repair in the liver is a highly complex matter and the visible results of a disturbed balance between many factors may be similar in several cases although the preceding sequence of events may have been very different. The mechanism of action of Senecio alkaloids is not yet known. It remains to be shown whether the hepatotoxic action is due to the parent alkaloid or to any of its metabolic products.

### SUMMARY.

Fifty-eight rats survived longer than 10 months of treatment with Senecio alkaloids from *S. jacobaea* Lin., retrorsine and isatidine. Forty-five of these cases showed changes in the liver ranging from hyperplasia to neoplasia. Metastases were found in 1 rat treated with isatidine.

Choline did not protect the liver from the action of isatidine.

One of us (R. S.) is greatly indebted to Professor J. W. Cook, F.R.S., for help and encouragement when the experiments were begun at the Chemistry Department, University of Glasgow ; to Professor C. M. Yonge, F.R.S., for kindly providing accommodation for the animals in the Department of Zoology ; and to Professor F. L. Warren, Chemistry Department, University of Natal, Pietermaritzburg, for a generous gift of pure isatidine and retrorsine. Her thanks are also due to Miss E. P. McLaren for technical assistance and excellent care of the animals. This work has been supported by a grant from the British Empire Cancer Campaign.

### REFERENCES.

Barger, G., and Blackie, J. J.—(1936) *J. Chem. Soc.*, *p.* 743.—(1937) *Ibid.*, p. 584.
Buckley, J. J., Buckley, S. M., and Snipes, A. E.—(1951) *Johns Hopk. Hosp. Bull.*, **89**, 218.
Christie, S. M. H., Kropman, M., Leisegang, E. C., and Warren, F. L.—(1949) *J. Chem. Soc.*, p. 1700.
Cook, J. W., Duffy, E., and Schoental, R.—(1950) *Brit. J. Cancer*, **4**, 405.
Copeland, D. H., and Salmon, W. D.—(1946) *Amer. J. Path.*, **22**, 1059.
Davidson, J.—(1935) *J. Path. Bact.*, **40**, 285.
Leisegang, E. C., and Warren, F. L.—(1950) *J. Chem. Soc.*, p. 702.
Opie, E.—(1944) *J. exp. Med.*, **80**, 231.
Steyn, D. G.—(1952) *S. Afr. med. J.*, **26**, 139.